# Tubular immunostimulating complex based on cucumarioside A_2_-2 and monogalactosyldiacylglycerol from marine macrophytes

**DOI:** 10.1186/1477-3155-9-35

**Published:** 2011-09-02

**Authors:** Eduard Y Kostetsky, Nina M Sanina, Andrey N Mazeika, Alexander V Tsybulsky, Natalia S Vorobyeva, Valery L Shnyrov

**Affiliations:** 1 Department of Biochemistry and Biotechnology, Far Eastern Federal University, Sukhanov St., 8, 690650 Vladivostok, Russia; 2 Departmento de Bioquímica y Biología Molecular, Universidad de Salamanca, plza Doctores de la Reina, 37007 Salamanca, Spain

## Abstract

**Background:**

There is an urgent need to develop safe and effective adjuvants for the new generation of subunit vaccines. We developed the tubular immunostimulating complex (TI-complex) as a new nanoparticulate antigen delivery system. The morphology and composition of TI-complexes principally differ from the known vesicular immunostimulating complexes (ISCOMs). However, methodology for the preparation of TI-complexes has suffered a number of shortcomings. The aim of the present work was to obtain an antigen carrier consisting of triterpene glycosides from *Cucumaria japonica*, cholesterol, and monogalactosyldiacylglycerol from marine macrophytes with reproducible properties and high adjuvant activity.

**Results:**

The cucumarioside A_2_-2 - cholesterol - MGalDG ratio of 6:2:4 (by weight) was found to provide the most effective formation of TI-complexes and the minimum hemolytic activity *in vitro*. Tubules of TI-complexes have an outer diameter of about 16 nm, an inner diameter of 6 nm, and a length of 500 nm. A significant dilution by the buffer gradually destroyed the tubular nanoparticles. The TI-complex was able to increase the immunogenicity of the protein antigens from *Yersinia pseudotuberculosis *by three to four times.

**Conclusions:**

We propose an optimized methodology for the preparation of homogeneous TI-complexes containing only tubular particles, which would achieve reproducible immunization results. We suggest that the elaborated TI-complexes apply as a universal delivery system for different subunit antigens within anti-infectious vaccines and enhance their economic efficacy and safety.

## Background

Immunostimulating complexes (ISCOMs) are a commonly known adjuvant that represents a supramolecular combination of saponins from *Quillaja saponaria*, cholesterol, and phosphatidylcholine. It has been shown that ISCOMs display high adjuvant activity against a broad range of bacterial and viral antigens [1-5]. However, the side effects of ISCOMs are their toxicity, caused by the presence of the hemolytic saponins of *Q. saponaria *[2,6], a consistently insufficient adjuvant activity, and the absence of a satisfactory method of preparing them for industrial applications [6-9]. The development of the ISCOMATRIX™ adjuvant, based on purified saponin fractions from Quil A substantially overcomes these shortcomings [1,10]. However, we proposed principally new biologically active components, glycoglycerolipids and triterpene glycosides isolated from marine macrophytes and invertebrates, respectively, to modify and optimize ISCOM vehicles for microbial antigens. The immunologically inert phospholipid phosphatidylcholine (PC) of ISCOMs was replaced by glycolipid monogalactosyldiacylglycerol (MGalDG) from marine macrophytes (algae and seagrasses) [11]. Subsequent substitution of the sum of saponins from *Q. saponaria *by triterpene glycosides from sea cucumbers, possessing immunostimulating activity, resulted in a tubular superstructure of the complexes (TI-complexes) instead of the vesicular one of classical ISCOMs [12]. Based on these results, we developed a prototype of TI-complex consisting of the sum of monosulfated triterpene glycosides from *Cucumaria japonica*, cholesterol, and MGalDG from marine macrophytes with a component weight ratio of 3:2:6, respectively. To obtain an antigen carrier, we further developed a method [13] based on that proposed earlier by Copland *et al. *[7]. Biological tests of the TI-complex prototype revealed that it had adjuvant activity [14-16].

However, these pilot preparations of TI-complexes appeared to be heterogeneous with a large amount of source material (amorphous phase of MGalDG + cholesterol), and intermediate structures apart from the complex particles. Perhaps this was a cause of non-reproducible and often contradictory immunization results. The presence of intermediate structures was also characteristic of classical ISCOM preparations obtained by methods of dialysis [17], the hydration of lipid films [7], and the injection of diethyl ether [9]. The aim of the present work was to obtain an antigen carrier based on triterpene glycosides from *C. japonica*, cholesterol, and MGalDG from marine macrophytes with reproducible properties and high adjuvant activity. The practical part of the work is protected by Application № 2010122159/10(031466) for Patents of the Russian Federation [18].

## Results and Discussion

### Immunological properties of the prototype TI-complex

**Figure 1:**
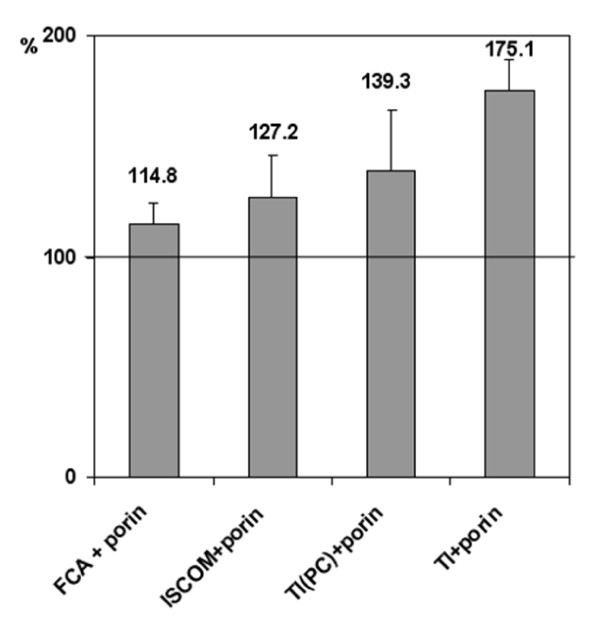
**Effect of different adjuvants on relative antibody response to porin YompF**. Along the ordinate, percentages with respect to the value for an anti-porin reference serum (serum of mice immunized with pure porin). Along the horizontal axis, the experimental group of animals immunized with: FCA+porin - porin mixed with Freund's complete adjuvant (Sigma); ISCOM+porin - porin incorporated in ISCOMs, prepared as described [11]; TI(PC)+porin - porin incorporated in TI-complex consisting of a cucumarioside A_2_-2, cholesterol and PC from egg yolk at weight ratio of 3:2:6; TI+porin - porin incorporated in TI-complexes consisting of a cucumarioside A_2_-2, cholesterol and MGalDG from *Ulva fenestrata *at weight ratio of 3:2:6. Dose of antigen (denaturated monomeric porin YompF) - 10 μg/mouse.

**Figure 2:**
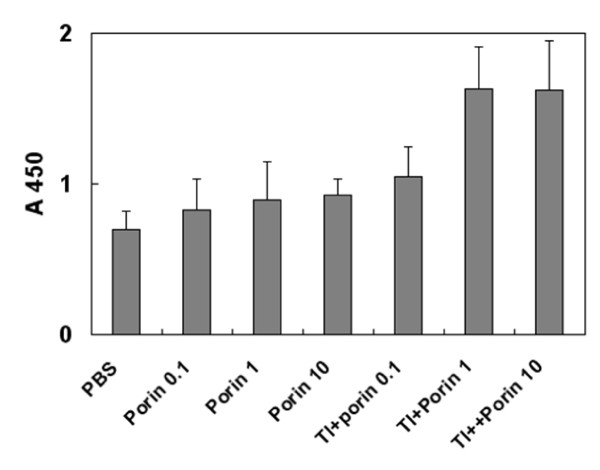
**Effect of TI-complex on the content of anti-porin antibodies at different doses of porin YompF**. Along the ordinate, optical density at 450 nm. Along the horizontal axis, experimental groups of animals immunized with: PBS - phosphate-buffered saline, Porin 0.1/1/10 - pure denaturated monomeric porin YompF at a dose of 0.1/1/10 μg/mouse, TI+porin 0.1/1/10 - denaturated monomeric porin YompF (a dose - 0.1/1/10 μg/mouse, respectively) incorporated in TI-complexes consisting of a cucumarioside A_2_-2, cholesterol and MGalDG from *Ulva fenestrata *at weight ratio of 3:2:6.

TI-complexes consisting of the sum of monosulfated triterpene glycosides from *Cucumaria japonica*, cholesterol, and MGalDG from marine macrophytes with a component weight ratio of 3:2:6 was used as prototype. It was observed that the thermally denatured monomeric porin from *Yersinia pseudotuberculosis *was effectively (not less than 95%) incorporated into TI-complex [15]. Immunization of mice with this protein antigen in TI-complexes provided stronger humoral immune response than one with porin in ISCOMs and Freund's complete adjuvant (Figure 1). Interestingly, the substitution of non-lamellar MGalDG for lamellar-prone PC in TI-complexes resulted in the lower immune response compared with the original TI-complex. Immunization with pure porin at doses of 0.1-10 μg/mouse did not reveal dose-dependent immune response, which was probably related to the immunosuppressive effect of porin at doses above 0.1 μg/mouse. The incorporation of antigen in TI-complex resulted in an increase in immune response only at these high doses, which may have been due to the deposition of antigen and the decrease in its immunosuppressive action (Figure 2).

**Figure 3:**
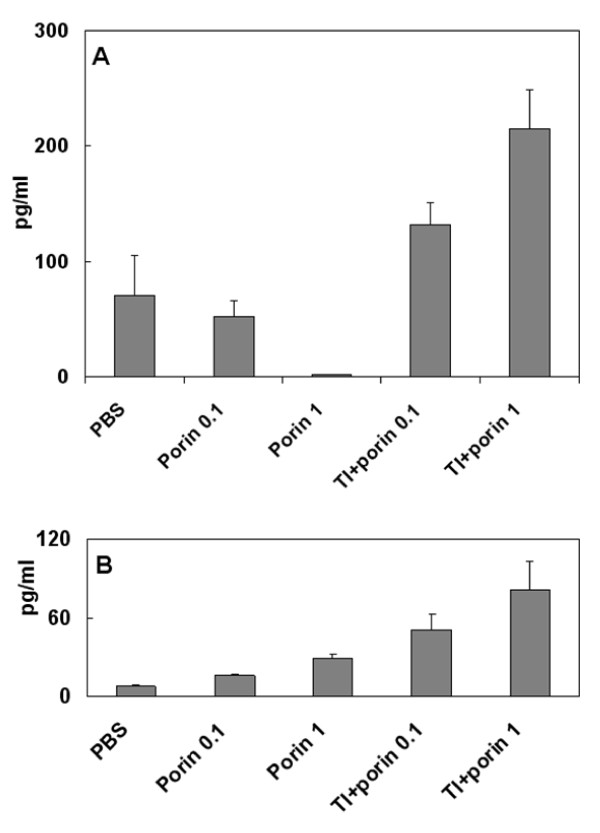
**Effect of TI-complexes on concentration of INF-γ (A) and IL-1β (B) at different doses of porin YompF**. Experimental groups of animals immunized with: PBS - phosphate-buffered saline, Porin 0.1/1 - pure denaturated monomeric porin YompF at dose of 0.1/1 μg/mouse, TI+porin - denaturated monomeric porin YompF (a dose - 0.1 or 1 μg/mouse, respectively) incorporated in TI-complexes consisting of a cucumarioside A_2_-2, cholesterol and MGalDG from *Ulva fenestrata *at weight ratio of 3:2:6.

The pure porin at doses of 0.1 and 1 μg/mouse also inhibited the production of INF-γ, which is a classical Th1 cytokine [19] (Figure 3A). The introduction of porin incorporated in TI-complexes resulted in the substantial enhancement of the INF-γ concentration. Concentration of another proinflammatory cytokine, IL-1β, [20] also increased (about three-fold) in immunization results with the same antigen incorporated in TI-complexes compared with pure porin (Figure 3B).

These results showed the bioavailability of TI-complexes as a new type of promising adjuvant carrier for antigens, although adjuvant activity of TI-complexes with a component weight ratio of 3:2:6 was not sufficiently high.

### The Choice of optimum composition for the TI-complex formation

We obtained chromatographically pure cucumarioside A_2_-2 from the sum of the triterpene glycosides of *C. japonica *and identified its structure by ^13^C NMR [21]. It is advantageous to use pure cucumarioside A_2_-2 rather than the sum of monosulfated triterpene glycosides, because it exhibits the highest immunostimulatory activity. Thus, the possible inclusion of immunosuppressive di- and tri-sulfated triterpene glycosides of *C. japonica *[22] in TI-complexes is avoided.

Energy-filtered transmission electron microscopy (EFTEM) was applied to unstained samples to investigate the homogeneity of the preparations of TI-complexes prepared with pure cucumarioside A_2_-2. The structure of the particles of the TI-complex was studied in samples that were negatively stained by phosphotungstic acid.

**Figure 4:**
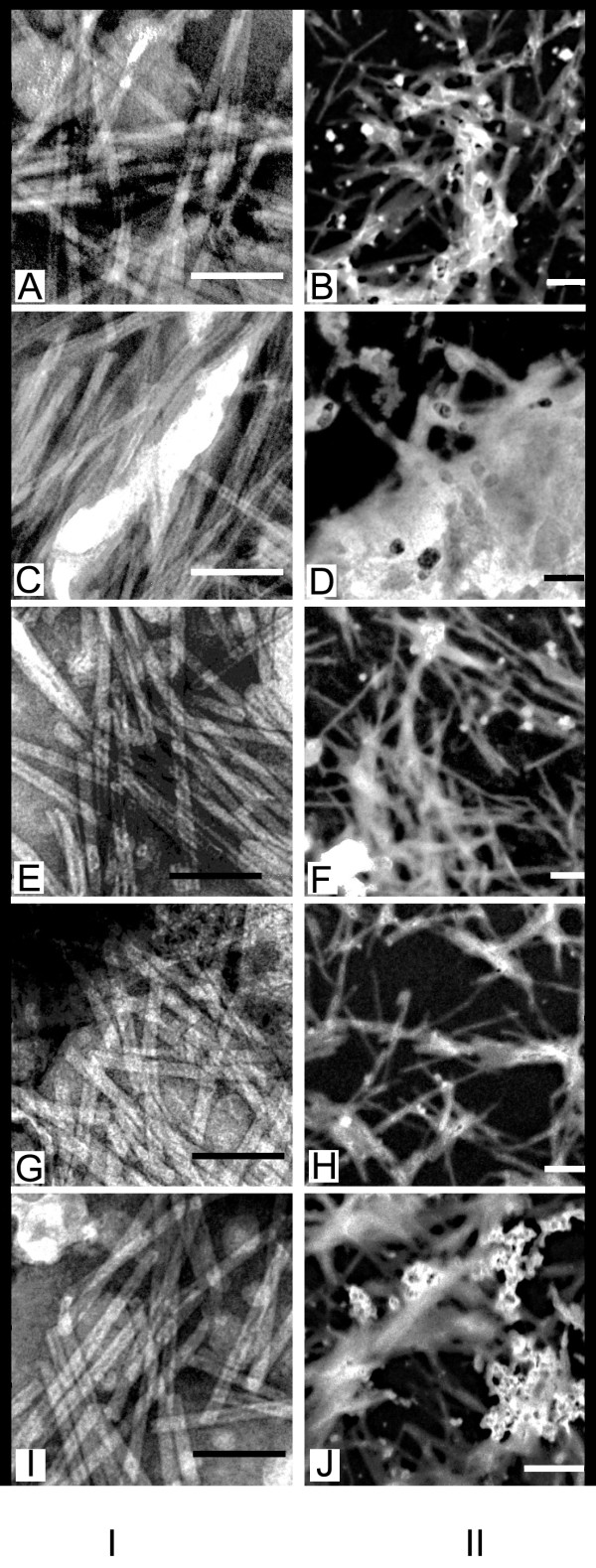
**Electron micrographs of the TI-complexes with different weight ratio of the components**. The ratios of cucumarioside A_2_-2 - cholesterol - MGalDG: A, B - 3:2:6; C, D - 1.5:2:6; E, F - 6:2:6; G, H - 6:2:4; I, J - 6:2:10. I - TEM micrographs of negatively stained samples, bar is 100 nm. II - EFTEM micrographs of unstained samples, bar is 200 nm.

Micrographs of negatively stained samples (Figure 4A) of the prototype TI-complex cucumarioside A_2_-2 - cholesterol - MGalDG, with a component weight ratio of 3:2:6, indicated the presence of typical tubular nanoparticles [13]. However, the unstained samples contained a substance that was not included in the tubular particles, probably representing excess MGalDG and cholesterol (Figure 4B). The two-fold smaller proportion of cucumarioside A_2_-2 in the cucumarioside A_2_-2 - cholesterol - MGalDG ratio (1.5:2:6) led to a sharp increase in the amount of a substance not included in the tubular particles (Figure 4D), whereas the negatively stained samples displayed a large number of typical tubular structures (Figure 4C). The two-fold increased (6:2:6) presence of cucumarioside A_2_-2 in the cucumarioside A_2_-2 - cholesterol - MGalDG ratio led to a more homogeneous system than in the prototype (Figure 4F), while the tubular structure of the particles persisted (Figure 4E). A further reduction in the MGalDG of cucumarioside A_2_-2 - cholesterol - MGalDG ratio to 6:2:4 led to the formation of a homogeneous system containing only particles with a typical tubular structure (Figure 4G) and it contained almost no extraneous substances (Figure 4H). The increase of MGalDG in the system up to 6:2:10 led to the appearance of a substance not included in the tubular structures (Figure 4J) in comparison with the cucumarioside A_2_-2 - cholesterol - MGalDG ratio of 6:2:6, while the tubular structure persisted (Figure 4I).

Thus, a cucumarioside A_2_-2 - cholesterol - MGalDG ratio of 6:2:4 (Figures 4G, H) prevented the formation of any particle other than the tubular type. This weight ratio of the components of the TI-complex was equimolar.

### Hemolytic activity of TI-complex

Study of the hemolytic activity of TI-complex preparations (Table 1) revealed that the cucumarioside A_2_-2 - cholesterol - MGalDG complex with a ratio of 6:2:4 showed minimum hemolytic activity. However, increasing proportions of MGalDG in the TI-complex were accompanied by an increase in its hemolytic activity, because this glycolipid alone showed its own hemolytic activity.

**Table 1:** Hemolytic activity (concentration causing 50% hemolysis of red blood cells - HD50) of TI-complexes and their components

Individual components of TI-complex	TI-complexes with different ratios of cucumarioside A_2_-2 - cholesterol - MGalDG	HD50, μg/ml
Cucumarioside A_2_-2		0.8
Saponins of *Quillaja saponaria*		25.0
MGalDG from *Zostera marina*		44.2
MGalDG from *Sargassum pallidum*		47.6
	6-2-15^a)^	13.6
	6-2-15^b)^	47.6
	3-2-6 ^a)^	43.0
	3-2-6 ^b) ^	60.8
	6-2-4 ^a)^	71.9
	6-2-4 ^b)^	109.2

^a) ^MGalDG from *Zostera marina*; ^b) ^MGalDG from *Sargassum pallidum*

### Optimal dilution of the TI-complex preparations

**Figure 5:**
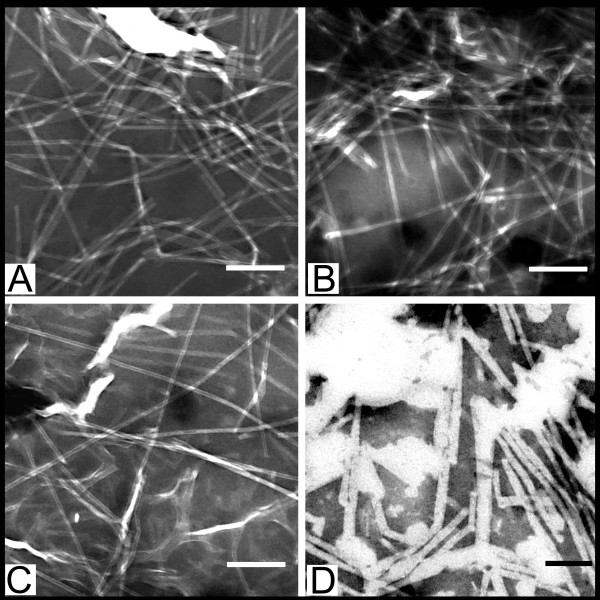
**Destruction of the TI-complex when diluted with phosphate buffer**. The weight ratio of cucumarioside A_2_-2 - cholesterol - MGalDG was of 3:2:6. A - An initial complex, scale bar: 200 nm; B - 5-fold diluted, scale bar: 200 nm; C - 10-fold diluted, scale bar: 200 nm; D - 75-fold diluted, scale bar: 100 nm. Negative staining.

Investigation of the stability of the TI-complexes revealed that dilution of the original preparations of the complex prototype with PBS led to a gradual destruction of the tubular nanoparticles and increased the amount of substance that was not included in the tubular structures (Figure 5). The five-fold dilution did not result in initial signs of destruction of the complex (Figure 5B), whereas the 75-fold dilution led to an almost complete destruction of the TI-complex (Figure 5D). This seems to be responsible for the low reproducibility of the immunization results by the prototype of the TI-complex [13,14], since the original preparation of the complex was diluted 75 times with phosphate buffer at immunization.

The TI-complex with a cucumarioside A_2_-2 - cholesterol - MGalDG component weight ratio of 6:2:4 diluted 10-folds with phosphate buffer was selected for the biological tests. The volume of the preparation for immunization was 10 μl per mouse. A further decrease in the dilution of the TI-complex in order to reduce its destruction was impossible, since the injection of less than 10 μl is technically difficult.

### Immunostimulating activity of TI-complex

**Figure 6:**
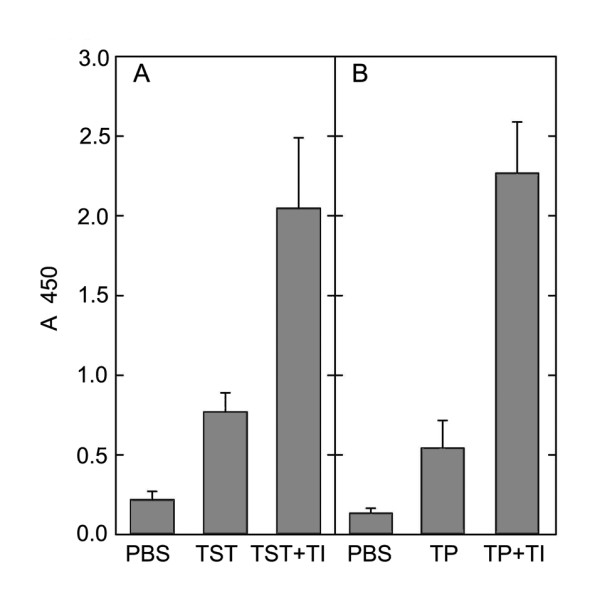
**Content of specific antibodies to the thermostable toxin (A) and YompF (B) from *Yersinia pseudotuberculosis***. Along the ordinate, optical density at 450 nm. Along the horizontal axis, the experimental group of animals immunized with: PBS - phosphate-buffered saline, TST - pure thermostable toxin, TST + TI - thermostable toxin in the TI-complex, TP - trimeric form of YompF, TP + TI - trimeric form of YompF in the TI-complex. TI-complexes comprised of a cucumarioside A_2_-2, cholesterol and MGalDG from *Ulva fenestrata *at weight ratio of 6:2:4.

The antibody response is the main indicator of adjuvant properties of any immunostimulating complexes or substances [23]. Biological tests in mice showed that the pore-forming YompF-protein from the outer membrane of *Y. pseudotuberculosis *cells in the TI-complex was significantly more immunogenic than when in pure form, as observed in a four-fold increase in optical density in ELISA for the trimeric forms of YompF, incorporated into the TI- complex (Figure 6A).

To confirm the adjuvant activity of the TI-complex, thermostable toxin (TST) from the same bacterium was also used [24]. Experiments addressing the immunization of mice with TST revealed that the injection of TST at a dose of 0.1 μg per mouse in the 6:2:4 TI-complex resulted in a three-fold increase in optical density in ELISA in comparison with the free antigen, indicating a significant adjuvant effect of the TI-complex with respect to TST (Figure 6B).

## Conclusions

In this work, we have determined the optimum ratio of components (cucumarioside A_2_-2, cholesterol and MGalDG) to form maximally homogeneous TI-complexes with minimal toxicity. The evidence of the complex destruction at the high dilution is also important to achieve reproducible immunization results. The data from biological tests revealed that the TI-complex can serve as a powerful adjuvant for subunit bacterial antigens.

## Methods

### Cucumarioside A_2_-2 isolation

Cucumarioside A_2_-2 was isolated by a modified method [21,22]. The body wall of the holothurian *Cucumaria japonica *(3 kg) was extracted twice for 3 h with an equal volume of 50% ethanol under conditions of boiling. The extracts thus obtained were combined and evaporated to dryness by the addition of 20% n-butanol.

80 g of the resulting extract was dissolved in 1.5 L of distilled water and the sum of triterpene glycosides and sterols was obtained by reverse phase chromatography using a column with 0.5 kg of Polichrom-1 (fraction of 0.5-1 mm) purchased from "Biolar", Latvia. The column length to diameter ratio was 5:1. The column was washed with distilled water up to a negative reaction for the chloride ion. The sum of triterpene glycosides and sterols was eluted with 50% ethanol. The yield of the substances was monitored by TLC on silica gel with chloroform-ethanol-water at 100:100:17 (v/v). The plates were sprayed with a solution of 10% sulfuric acid in methanol, followed by heating at 150°C until dark spots appeared.

For further removal of macromolecular impurities, the fraction was evaporated to dryness (3 g), and then repeatedly extracted with systems of chloroform-ethanol-water 100:75:10 and 100:100:17 (v/v). The extracts were combined, evaporated to dryness (1.5 g), and re-dissolved in a minimum volume of chloroform-ethanol-water at 100:100:17 (v/v).

Sterols and sterol sulfates were separated on a silica gel (100/160 μm fraction, Chemapol, Czech Republic) column (the ratio of length to diameter was 10:1) equilibrated with chloroform-ethanol 3:1 (v/v). The sorbent-substance ratio was 100:1. The column was eluted with chloroform-ethanol 3:1 (v/v). After separation of the sterols (750 mg), the triterpene glycosides were eluted with chloroform-ethanol-water at 100:100:17. The yield of substances was monitored by TLC as described above. Fractions corresponding to the sum of monosulfated triterpene glycosides were pooled and evaporated to dryness (450 mg). Then, the sum of monosulfated triterpene glycosides was separated on a silica gel (40-63 μm, Merck, Germany) column (length to diameter ratio 10:1), equilibrated with chloroform-ethanol-water 100:100:17 (v/v). The sorbent - triterpene glycoside ratio was 150:1. The substances were eluted with the same system. Fractions corresponding to cucumarioside A_2_-2 (control by TLC using a standard of cucumarioside A_2_-2) were combined and evaporated to dryness (200 mg).

Cucumarioside A_2_-2 was isolated by preparative reverse phase HPLC-MS followed by HPLC-MS on silica gel. This was performed with a SHIMADZU LC-8A chromatograph. Substance output was detected in negative ion mode at 3 kV using an HPLC LCMS - 2010EV mass spectrometer. Reverse phase chromatography was performed on a Sim-pack PREP-ODS (20 × 250 mm) column. 1.5 ml of an aqueous solution of the sum of triterpene glycosides at a concentration of 10 mg/ml was injected into the column and eluted with a methanol-water gradient of 70% - 20 min; 75% - 20 min, and 90% - 40 min at a rate of 10 ml/min. The fraction corresponding to ion m/z of 1299 was selected. HPLC was performed on a PREP-sil silica gel Sim-pack column (20 × 250 mm). 2 ml of the triterpene glycosides at a concentration of 5 mg/ml in a 73.2:22.95:3.8 (v/v) chloroform-methanol-water system was injected into column and eluted with the same system at a rate of 10 ml/min. The selected fraction, corresponding to ion m/z of 1299, was a pure cucumarioside A_2_-2 (80 mg).

The structure of purified cucumarioside A_2_-2 in deuteropyridine was identified by ^13^C NMR spectroscopy on a Brucker-500 spectrometer. The spectrum was identical to that previously published for cucumarioside A_2_-2 [22,25].

### Isolation of monogalactosyldiacylglycerol

MGalDGs from marine macrophytes were isolated and their fatty acid analysis was performed as described previously [26]. Chromatographically pure MGalDG was dissolved in chloroform and stored at -20°C in a light-proof container.

### Preparation of TI-complexes

To obtain the TI-complex with a cucumarioside A_2_-2-cholesterol-MGalDG component ratio of 6:2:4, 5 mg of MGalDG was dissolved in 1 ml of chloroform; 5 mg of cholesterol was dissolved in 1 ml of chloroform; and 4 mg of cucumarioside A_2_-2 was dissolved in 1 ml of distilled water. Then, a 66-μl solution of MGalDG and a 33-μl solution of cholesterol were evaporated to dryness under a stream of air at a temperature of 60°C. A 125-μl solution of cucumarioside A_2_-2 was added to this dry residue. Then, 375 μl of PBS, pH 7.2, was added to this mixture, adjusting the concentration of MGalDG and cholesterol to 1 mg/ml. The suspension thus obtained was sonicated for 5 min on a SONOPULS Ultrashall-Homogenizatoren HD 2070 (Germany) ultrasonic disintegrator at 10% maximum power (0.7 sec - work; 0.3 sec - interval). After sonication, the preparation was left to stand at room temperature for 2 h.

To obtain the TI-complexes with other component ratios, the volume of the aliquots of the components was increased or decreased. However, the final volume of the preparation was always 0.5 ml and the total concentration of MGalDG and cholesterol was 1 mg/ml.

To obtain the YompF-containing TI-complex with 0.1 μg of protein per 1 μg of cucumarioside A_2_-2, 100 μl of the lipid-saponin complex was collected immediately after sonication and combined with a 10-μl YompF solution in PBS, pH 7.2, at a concentration of 1 mg/ml. This mixture was vortexed for 1 min. Then, the preparation was left to stand at room temperature for 2 h.

The complex containing thermostable toxin (TST) was prepared in a similar way to the complex containing YompF. To obtain the complex with 0.1 μg TST per 1 μg of cucumarioside A_2_-2, 75 μl of lipid-saponin complex was collected and added to 25 μl of the TST solution in PBS, pH 7.2, at a concentration of 0.3 mg/ml.

To study immunological properties of the TI-complex prototype, the complex of monosulfated triterpene glycosides from *C. japonica*, cholesterol and MGalDG from *Ulva fenestrata *(or PC from egg yolk) were mixed at the weight ratio of 3:2:6. Then denaturated monomeric porin from *Y. pseudotuberculosis *at a respective dose (0.1, 1 or 10 μg of protein per 1 μg of glycosides) was added to TI-complexes. The resulted mixture was sonicated by ultrasonic disintegrator SONOPULS Ultrashall-Homogenizatoren HD 2070 (Germany) at 10% maximum power (0.7 sec - work; 0.3 sec - interval) [13-15]. Porin-containing ISCOMs were prepared as described [11].

Trimer and denatured monomer forms of YompF were isolated from *Y. pseudotuberculosis *(strain 598, I serovar) by researchers at the Laboratory of the Molecular Bases of Antibacterial Immunity, PIBOC, FEB RAS following a method [27]. Thermostable toxin [24] was provided by collaborators at Laboratory of the Molecular Bases of Bacterial Pathogenicity at the Research Institute of Epidemiology and Microbiology, SB RAMS.

### Transmission electron microscopy

Transmission electron microscopy (TEM) and energy-filtered transmission electron microscopy (EFTEM) were performed with Libra-120 microscope (Zeiss, Germany) at an accelerating voltage of 120 kV. Each suspension of the TI-complexes was investigated by TEM on the negatively stained samples and by EFTEM on the unstained samples. Copper grids of 300-400 mesh with a formvar substrate were used to prepare the samples [28].

To prepare the samples, 1-2 μl of a TI-complex suspension was placed on a grid and then dried in a stream of warm air. The dried grid was washed three times with 3 μl of distilled water, removing the excess with filter paper. To prepare the unstained samples, the washed grids were dried in a stream of warm air. To obtain the negative stained samples, 1 μl of a 0.5% solution of phosphotungstic acid, pH 7.2, was placed on the washed grid and then dried in a stream of warm air.

### Hemolytic activity

The hemolytic activity of the TI-complexes was determined by a direct hemolysis reaction. A 0.5% suspension of human erythrocytes in 0.01 M PBS, pH 7.2, was used as a test system. 0.1 ml of the erythrocyte suspension was added per well onto a 96-well plate. Then, 0.1 ml of TI-complexes at several dilutions was added in 3 repeats. The mixtures were incubated at 37°C for 1 h and centrifuged at 2000 rpm for 10 min. The supernatant was transferred to the wells of a 96-well flat-bottomed plate. Photometry was performed at 540 nm using an ELX808IU photometer (BioTek Instr., USA) to determine the minimum effective concentration of the substance that caused hemolysis in 50% of red blood cells (HD50).

### Adjuvant activity

Laboratory mice (Balb/c strain) with weight of 18-21 g were used. Animals were keeping in vivarium at standard conditions with unlimited access to food and water according with the rules accepted by European Convention for the Protection of Vertebrate Animals used for Experimental and Other Scientific Purposes (Strasbourg, 1986). Research was carried out according with the rules of the proper laboratory practice (GLP), Order of Ministry of Health of Russian Federation №267, 19.06.2003 "About the approval of rules for laboratory practice", Guide on experimental (pre-clinical) study of new pharmacological substances" (2005).

The TI-complex was injected at a dose of 1 μg of cucumarioside A_2_-2 and 0.1 μg of YompF or thermostable toxin per mouse. Immediately before injection, the original preparations of antigen-containing TI-complexes were diluted with PBS, and 10 μl of this solution was injected subcutaneously into the thighs of the animals.

To study the immunological properties of the TI-complex prototype, pure porin and porin with adjuvants (TI-complex prototype, ISCOMs or Freund's complete adjuvant (Sigma)) were injected intraperitoneally into mice at doses of 0.1, 1, and 10 μg/mouse in 100 μl of PBS.

The preparations were administered on the 1^st ^and 7^th ^day of the experiment. Sera were collected on day 21. In all experiments each experimental group consisted of 10 animals.

Specific antibodies in blood serum were determined by the ELISA method. The relative concentration of antibodies was expressed in optical density units at a wavelength of 450 nm (dilution of serum - 1/100).

Cytokines (INF-γ and IL-1β) were determined in the blood serum of mice in the respective groups by non-competitive ELISA using BD OptEIA™ Mouse ELISA kits (BD, USA). Absorption was recorded with an Elx808IU microplate photometer, Biotek Instr., USA, at a wavelength of 450 nm.

## Competing interests

Authors of the present work are currently applying for patent relating to the content of the manuscript (Application № 2010122159/10(031466) for Patent of Russian Federation). We have not received reimbursements, fees, funding, or salary from an organization (Far Eastern Federal University) that has applied for patent relating to the content of the manuscript.

## Authors' contributions

EYK and NMS planned and coordinated the present research. NMS also proposed the idea of immunostimulating complexes based on lipids from marine hydrobionts. ANM prepared TI-complexes, controlled their structure, stability and hemolytic activity. AVT immunized animals and analyzed the content of specific antibodies. NSV isolated cucumarioside A_2_-2 and monogalactosyldiacylglycerol. VLS identified cucumarioside A_2_-2. All authors read and approved the final manuscript.
